# Insights of Platinum Drug Interaction with Spinel Magnetic Nanocomposites for Targeted Anti-Cancer Effect

**DOI:** 10.3390/cancers15030695

**Published:** 2023-01-23

**Authors:** Dana Almohazey, Vijaya Ravinayagam, Widyan Alamoudi, Sultan Akhtar, H. Dafalla, Hind Nasser AlSuwaidan, Shoruq. T. Almutairi, Hajer Saleh Alghamdi, Sukaina Ahmed Aldamen, M. A. Almessiere, A. Baykal, Ahmed A. Maarouf, B. Rabindran Jermy

**Affiliations:** 1 Department of Stem Cell Research, Institute for Research and Medical Consultations, Imam Abdulrahman Bin Faisal University, P.O. Box 1982, Dammam 31441, Saudi Arabia; 2 Deanship of Scientific Research & Department of Nano-Medicine Research, Institute for Research and Medical Consultations, Imam Abdulrahman Bin Faisal University, P.O. Box 1982, Dammam 31441, Saudi Arabia; 3 Department of Neuroscience Research, Institute for Research and Medical Consultations, Imam Abdulrahman Bin Faisal University, P.O. Box 1982, Dammam 31441, Saudi Arabia; 4 Department of Biophysics Research, Institute for Research and Medical Consultations, Imam Abdulrahman Bin Faisal University, P.O. Box. 1982, Dammam 31441, Saudi Arabia; 5 Core Research Facilities (CRF), King Fahd University of Petroleum and Minerals, Dhahran 31261, Saudi Arabia; 6 PharmD, College of Clinical Pharmacy, Imam Abdulrahman Bin Faisal University, P.O. Box 1982, Dammam 31441, Saudi Arabia; 7 Department of Pharmaceutics, College of Clinical Pharmacy, Imam Abdulrahman Bin Faisal University, Dammam 31441, Saudi Arabia; 8 Department of Nano-Medicine Research, Institute for Research and Medical Consultations, Imam Abdulrahman Bin Faisal University, P.O. Box 1982, Dammam 31441, Saudi Arabia; 9 Department of Physics, College of Science, Imam Abdulrahman Bin Faisal University, P.O. Box 1982, Dammam 31441, Saudi Arabia; 10 Department of Physics, Faculty of Basic Sciences, The German University in Cairo, New Cairo 13411, Egypt

**Keywords:** multifunctional, manganese ferrite, cisplatin, drug delivery, nanotherapeutics, density functional theory

## Abstract

**Simple Summary:**

Magnetic ferrite nanocomposite has drawn huge interest in nanomedicine in areas related to thermotherapy, cell labeling-tracking and magnetic resonance imaging. Manganese ferrite spinel is an interesting magnetic nanocomposite due to its superparamagnetic nature, strong T2 MRI contrast, low synthesis cost, and eco-friendliness. The present study investigated the suitability of two different nanocarriers: one with a silica base (MnFe_2_O_4_/silica), and another with a carbon base (MnFe_2_O_4_/Graphene oxide) for targeted cancer therapy. The phase, textural and morphological variation of the two different nanoformulations was examined using various physico-chemical techniques. Pegylated and as-such nanoformulations were studied in drug delivery and in vitro using cancerous and non-cancerous cell lines. Density functional theory was used to calculate the binding energies between cisplatin on single-silica or multi-layered graphene oxide. Immunofluorescence images were captured using c-caspase 3/7 and TEM analysis. MnFe_2_O_4_/silica/cisplatin nanocomposites was found be a better chemotherapeutic drug delivery option than MnFe_2_O_4_/GO/cisplatin nanocomposites.

**Abstract:**

In nanotherapeutics, gaining insight about the drug interaction with the pore architecture and surface functional groups of nanocarriers is crucial to aid in the development of targeted drug delivery. Manganese ferrite impregnated graphene oxide (MnFe_2_O_4_/GO) with a two-dimensional sheet and spherical silica with a three-dimensional interconnected porous structure (MnFe_2_O_4_/silica) were evaluated for cisplatin release and cytotoxic effects. Characterization studies revealed the presence of Mn^2+^ species with a variable spinel cubic phase and superparamagnetic effect. We used first principles calculations to study the physisorption of cisplatin on monodispersed silica and on single- and multi-layered GO. The binding energy of cisplatin on silica and single-layer GO was ~1.5 eV, while it was about double that value for the multilayer GO structure. Moreover, we treated MCF-7 (breast cancer cells) and HFF-1 (human foreskin fibroblast) with our nanocomposites and used the cell viability assay MTT. Both nanocomposites significantly reduced the cell viability. Pt^4+^ species of cisplatin on the spinel ferrite/silica nanocomposite had a better effect on the cytotoxic capability when compared to GO. The EC50 for MnFe_2_O_4_/silica/cisplatin and MnFe_2_O_4_/GO/cisplatin on MCF-7 was: 48.43 µg/mL and 85.36 µg/mL, respectively. The EC50 for the same conditions on HFF was: 102.92 µg/mL and 102.21 µg/mL, respectively. In addition, immunofluorescence images using c-caspase 3/7, and TEM analysis indicated that treating cells with these nanocomposites resulted in apoptosis as the major mechanism of cell death.

## 1. Introduction

Spinel ferrite with magnetic and electrical properties has been widely investigated in hyperthermia, medical diagnostics, cell labeling/tracking and several other biomedical device applications [1]. The use of magnetic nanocarriers has the potential to improve the targeted delivery of chemotherapeutic drugs, minimize the dosage and enhance patient adherence [2]. MnFe_2_O_4_ with a cubic inverse spinel (Fd3m) structure exhibits several attractive properties for biomedical applications, such as superparamagnetism, high MRI contrasting ability, aligning capability to external magnetic field, and stability in physiological conditions [3,4]. Magnetogel, an incorporated manganese ferrite nanoparticles into a dehydropeptide-derived hydrogel matrix, has been reported as a vehicle for anti-cancer drugs such as thienopyridine derivatives and curcumin. The gel with RGD peptide sequence (Arg-Gly-Asp) exhibited an intrinsic fluorescence and was found to be promising for thienopyridine derivative delivery [5]. The use of polyvinylpyrrolidone (PVP)-coated MnFe_2_O_2_ with hydrophilic properties has been studied for controlled drug delivery. These particles demonstrated a superparamagnetic property with a uniform particle dispersion and narrow size distribution. The in vitro experiments of these nanoparticles highlighted a negligible cytotoxicity even at high concentrations. Moreover, these nanoparticles showed an excellent drug loading capacity with doxorubicin and exhibited an interesting pH-dependent release behavior [6]. Similarly, multifunctional mesoporous silica-coated superparamagnetic manganese ferrite has been prepared and assessed for their performance in the targeted drug delivery of doxorubicin. In addition, they showed a better drug release at lysosomal pH (pH 5.6), high biocompatibility and were significantly effective in reducing the cell viability of a cancerous cell line with insignificant effects on a normal cell line [7].

Graphite and related allotropes including GO in reduced and oxidized forms and carbon nanotubes with different metal oxides have been extensively applied for biomedical applications, such as drug delivery [8], biosensors [9] and cancer theranostics [10]. The main reason for such applications is attributed to the unique textural, structural, and geometrical features, such as a high surface area, electrical and thermal conductivity [11], biocompatibility and reduced toxicity [12]. Graphene oxide has been recently investigated as a multifunctional platform for drug delivery, sensor, and imaging applications [13]. We have reported the spherical silica loaded with spinel ferrite CuFe_2_O_4_ and SPIONs/Cubic structured SBA-16 for cisplatin delivery application [14,15]. However, until now, a characteristic comparison between manganese ferrite on silica or GO for cisplatin drug release and their anti-cancer activity is not reported. In this study, two nanocomposites: manganese ferrite/spherical silica (MnFe_2_O_4_/silica) and manganese ferrite/graphene oxide (MnFe_2_O_4_/GO) were prepared and characterized. The cisplatin release behavior was explored along with cytotoxic activity. Furthermore, the density functional theory was used to calculate the binding energies between cisplatin and both silica and GO.

## 2. Material and Methods

Graphene oxide (GO), Mn(NO_3_)_2_.3H_2_O, Fe(NO_3_)_3_.9H_2_O, and cisplatin were purchased from Sigma Aldrich, while spherical silica was obtained from Superior silica, USA. 

### 2.1. Preparation of 30% MnFe_2_O_4_/silica and GO

The manganese ferrite-based silica and GO were prepared by physically mixing manganese nitrate trihydrate (0.14 g), iron nitrate nonahydrate (1.05 g) and 1.4 g of nanocarrier (silica or GO) for 30 min using mortar and pestle. The homogenized powder was then collected and calcined (850 ℃/6 h) for further modification. 

### 2.2. Cisplatin loading on MnFe_2_O_4_/silica and MnFe_2_O_4_/GO

The chemotherapeutic drug cisplatin (7.5 mg) and nanocarrier (150 mg) with a drug/nanocarrier ratio of 0.05 was mixed in a normal saline solution overnight in an ice cool condition. Then, the mixture was filtered, dried at room temperature and collected. The collected solution was analyzed at specific wavelength of 208 nm using UV-visible spectroscopy. The entrapment efficiency (EE %) and the loading capacity (LC %) of the two formulations were calculated using the following formula:

% Entrapment efficiency = (Cisplatin_initial_ − Cisplatin_supernatant_)/Cisplatin_initial_ × 100% (1)

% Loading capacity = (Cisplatin_initial_ − Cisplatin_supernatant_)/Nanocarrier × 100% (2)

The calculation revealed that MnFe_2_O_4_/silica had an EE % of 87% and LC % of 4.3%, while MnFe_2_O_4_/GO had an EE % of 92% and LC % of 4.6%. Both samples were pegylated using lyophilization technique. For instance, 14 μL of PEG (MW = 400) was dissolved in 3 mL of distilled water. Then, 150 mg of MnFe_2_O_4_/silica/cisplatin or MnFe_2_O_4_/GO/cisplatin was added and allowed to stir for 24 h. Then, the sample was lyophilized.

### 2.3. Characterization Techniques

The MnFe_2_O_4_ phase on silica and GO was analyzed using high-angle XRD instrument (Miniflex 600, Rigaku, Tokyo, Japan). The textural features of two supports were measured using the N_2_ adsorption technique (ASAP-2020 plus, Micromeritics, Norcross, GA, USA). The morphological variations of manganese ferrite nanoparticles over the silica and GO support was investigated using transmission electron microscopy (TEM, JEM2100F, JEOL, Tokyo, Japan). The sample preparation procedure was described in our previous publication [14]. The superparamagnetic properties of the two composites were analyzed using vibrating sample magnetometer (VSM), LDJ electronics, 9600, Troy MI, USA. The ferrite nanoparticle chemical coordination was analyzed using DRS-UV-visible spectroscopy analysis (JASCO V-750, Tokyo, Japan). 

### 2.4. Drug Release Study

The drug release profile of MnFe_2_O_4_/silica, MnFe_2_O_4_/GO and pegylated nanoformulations were studied using dialysis memberane technique. Before the study, the membrane was activated by soaking in the normal saline solution at 37 °C. Then 30 mg of nanoformulation was dispersed in 50 mL of PBS solution (pH 5.6 and 7.4). At regular time intervals, 10 mL of solution was withdrawn, and cisplatin release was measured using UV-visible spectroscopy. The withdrawn solution was replaced with an equal volume of fresh PBS solution.

### 2.5. Computational Details

The density functional theory (DFT) calculations were performed using the Quantum Espresso plane waves package based on density functional theory [16]. We used the PBE generalized gradient approximation [17] with ultrasoft pseudopotentials with a kinetic energy cutoff of 45 Ry. A vacuum *z*-axis separation between 10 \AA and 13 \AA is maintained in all systems to prevent interactions between neighboring cells. Van der Waals interactions (vdW) were calculated within the semi-empirical DFT-D2 and the first principles vdW-DF methods [18]. All systems were structurally relaxed at the Gamma point until the total forces were less than 0.002 Ry/Bohr.

### 2.6. In Vitro Study

For testing the cytotoxic effects of our formulations, we used the human mammary adenocarcinoma (MCF7), human cervical adenocarcinoma (HeLa), human colorectal cancer (HCT116), human foreskin fibroblasts (HFF-1), and human embryonic kidney (HEK293) cell lines. Cells were maintained in DMEM (Dulbecco’s Modified Eagle Medium) (Gibco, life technologies) supplemented with 10% heat inactivated fetal bovine serum (HI-FBS) (Gibco, life technology), 1% Penicillin Streptomycin (100X- Gibco, life technology), and 1% MEM NEAA (MEM non-essential amino acids) (100X- Gibco, life technology). Cells were cultured at 37 °C under a humidified condition and 5% CO_2_. For cell viability assays, 20,000 cells/well were plated on 96-well plates, whereas for electron microscopy analysis, 700,000 cells/well were plated on 6-well plates. Cultured cells were changed to starve media (0.5% HI-FBS containing media) for 24 h before treatment.

#### 2.6.1. Cell Treatment

Cells were treated for 48 h with the following conditions: MnFe_2_O_4_, silica, graphene oxide (GO), cisplatin, MnFe_2_O_4_/silica/cisplatin, and MnFe_2_O_4_/GO/cisplatin. Treatment concentrations for silica, GO, MnFe_2_O_4_/silica/cisplatin, and MnFe_2_O_4_/GO/cisplatin were as follows: 0.025, 0.05, 0.1, and 0.5 mg/mL. We used drug loading experiment to determine the concentration of MnFe_2_O_4_ and cisplatin that were loaded into nanoparticles in groups: MnFe_2_O_4_/silica/cisplatin, and MnFe_2_O_4_/GO/cisplatin. These experiments showed that 0.045 mg of cisplatin was loaded in 1 mg of these nanocomposites. Thus, if the experimental concentration of MnFe_2_O_4_/silica/cisplatin was 0.025 mg/mL, then the actual cisplatin concentration is 0.001125 mg/mL. Furthermore, if the experimental concentration of MnFe_2_O_4_/silica/cisplatin was 0.025 mg/mL, then the actual MnFe_2_O_4_ in the nanocomposite is 0.0084 mg/mL. Therefore, the experimental concentrations for cisplatin were: 0.001125, 0.00225, 0.0045, and 0.0225 mg/mL. The experimental concentrations for MnFe_2_O_4_ were: 0.0084, 0.0168, 0.0336, and 0.168 mg/mL.

#### 2.6.2. Cell Viability MTT Assay

We used the cell viability 3-(4,5-dimethylthiazol-2-yl)-2,5-diphenyltetrazolium bromide (MTT) assay to test the nanoformulations’ cytotoxic effects. If cells are viable, they will reduce the yellow-colored MTT to the blue-colored formazan. The protocol was as follows: After the treatment duration, cells were washed with PBS and then the MTT (Sigma-Aldrich) solution was added to each well at a concentration of 0.5 mg/mL. The 96-well plate was incubated at 37 °C for three hours [19]. After that, a solubilizing solution (0.04 N HCl isopropanol) was added to dissolve the formazan crystals. The absorbance was measured at 570 nm using SYNERGY-neo2 BioTek ELISA plate reader. Each condition was performed in triplicate, which were then averaged. In addition, an “initial reading” was measured at 570 nm before the addition of MTT. The “initial reading” was subtracted from the “final reading” to eliminate unwanted interreference. Each condition was then compared to the no-treatment control. The following equation was used to calculate the % of cell viability:

% Cell Viability = (averaged sample read/averaged control read) × 100

#### 2.6.3. Transmission Electron Microscopy (TEM)

After treating the cells as mentioned above, they were detached from the wells with trypsin. Cells were centrifuged and 3% Glutaraldehyde was added to the pellet. After washing, 1% Osmium tetroxide was added to each sample followed by several washing steps. Cell pellets were dehydrated, and pure epoxy resin was added to each sample and embedded at 60 °C for 24 h. Finally, the embedded cell pellets were sectioned at 50 nm thickness and imaged under a transmission electron microscope.

#### 2.6.4. Immunofluorescent and Light Microscopy

Cells were plated on a chamber slide and treated as described above. After 48 h of treatment, cells were stained with Hoechst 33,342 (Thermo Scientific, cat. 62249, Waltham, MA, USA) at a concentration of 2 μg/mL for 20 min at 37 °C. After washing with PBS, cells were stained with CellEvent Caspase-3/7 (Invitrogen, cat. C10423, Paisley, UK) at a concentration of 5 μM for 30 min at 37 °C. Cells were then imaged using the Zeiss LSM 700 confocal microscope. Light microscopy images were taken using the Nikon Eclipse TS100. Immunofluorescent and light microscopy images were not taken from the same field but were taken from the same sample.

#### 2.6.5. Annexin V/Propidium Iodide Analysis

HCT116 cells were cultured in white clear bottom 96-well plates (Thermo Scientific, cat. 165306) at a density of 50,000 cells/well. Cells were treated for 6 and 24 h with 0.05 mg/mL of MnFe_2_O_4_/silica/cisplatin and MnFe_2_O_4_/GO/cisplatin and its equivalent concentration of cisplatin (0.00225 mg/mL for details, please check Section 2.6.1). After that, cells were stained with Annexin V (AV) (Invitrogen, cat. BMS147FI), Propidium Iodide (PI) (Invitrogen, cat. P3566), and Hoechst (Thermo Scientific, cat. 62249) as indicated by the manufacturer’s instructions. Then, the fluorescent intensity was measured using SYNERGY-neo2 BioTek ELISA plate reader. The excitation/emission wavelengths used for AV, PI, and Hoechst were as follows: 485/528, 530/590, and 360/460, respectively. The signal intensities of AV and PI were normalized to the Hoechst signal. As described before [20], our analysis was based on the following classification: live AV−/PI−; necrosis AV−/PI+; early apoptosis AV+/PI−; and late apoptosis (or secondary necrosis) AV+/PI+.

#### 2.6.6. Statistics

Cell viability assay data represent four independent experiments. The AV/PI analysis represents five independent experiments. Statistical analysis was performed using Prism 8 software (GraphPad). The analysis was performed using two-way ANOVA with Dunnett’s multiple comparison post hoc test. ** *p* < 0.01; *** *p* < 0.001; **** *p* < 0.0001 versus control. Error bars ± standard error of the mean (S.E.M).

## 3. Results and Discussion

### 3.1. Characterization Studies

**Figure 1:**
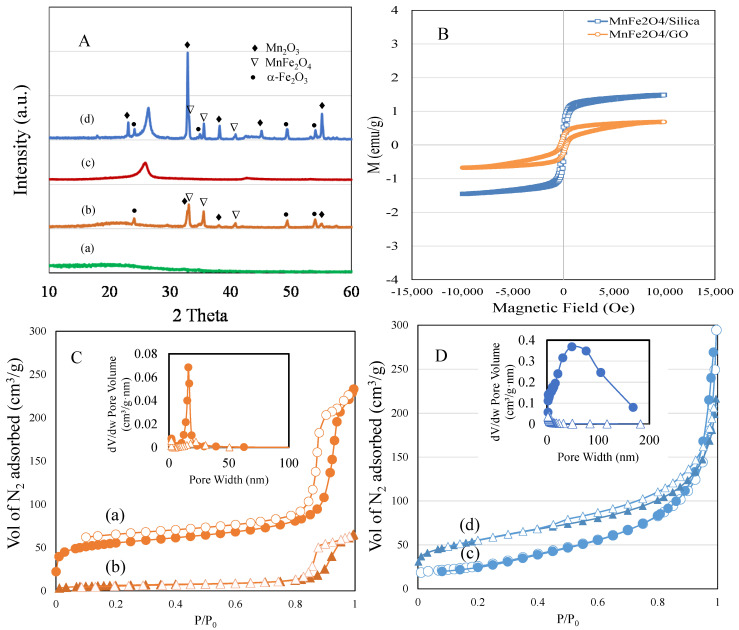
(**A**) (a–d). XRD powder patterns of: (a) silica; (b) MnFe_2_O_4_/silica; (c) GO; and (d) MnFe_2_O_4_/GO. (**B**) Vibrating sample magnetometer spectrum of MnFe_2_O_4_/silica and MnFe_2_O_4_/GO. (**C**) (a,b). BET adsorption–desorption isotherm and pore size distributions of: (a) silica; and (b) MnFe_2_O_4_/silica. (**D**) (c) GO; and (d) MnFe_2_O_4_/GO. Closed symbol represents adsorption and open one represents desorption.

Figure 1A shows the XRD patterns of (a) silica, (b) MnFe_2_O_4_/silica, (c) GO, and (d) MnFe_2_O_4_/GO. silica showed the amorphous diffraction peaks (2θ = 15–30°), while GO showed two peaks at 18.0° and 43.0°, which is characteristic of the amorphous nature of GO. XRD analysis of MnFe_2_O_4_/silica revealed the presence of crystalline cubic phase of MnFe_2_O_4_ over silica support. The sample exhibited distinct peaks at 18.0°, 32.9°, 35.6°, 43.6°, 53.2° and 55.1° corresponding to (111), (220), (311), (400), (422), and (511) planes. Samples from the silica support displayed an additional but a less intense peak corresponding to Mn_2_O_3_ that occurs at 32.9°. The peaks were accompanied with peaks of α-Fe_2_O_3_ at 24.2°, 49.4° and 54.1°. When the spinel was loaded over GO, an intense peak corresponding to Mn_2_O_3_ was observed at 32.9° followed by peaks of MnFe_2_O_4_. Compared to the silica support, the Mn_2_O_3_ phase was dominant over GO followed by manganese ferrite and α-Fe_2_O_3_. The presence of a mixed phase shows the instability of Mn^2+^ ions over GO support [21]. The decomposition reaction of spinel ferrite during the calcination process proposed to form such mixed phase, as carbon support tends to be less stable at such high calcination temperature.

The magnetic properties of MnFe_2_O_4_/silica and MnFe_2_O_4_/GO nanocomposites were analyzed by VSM (Figure 1B). The magnetic characteristics were influenced by the cation placement at the coordinative sites of A and B. In our case, MnFe_2_O_4_/silica showed superparamagnetic property indicating presence of antiparallel spins due to ferric cation at tetrahedral site with a saturation magnetization value of 1.5 emu/g. The nanocluster size on the pore walls of structured nanocarrier such as MCM-41 have been shown to influence the magnetic behaviour, such as superparamagnetic with small size nanocluster or ferromagnetization with large nanoclusters [22]. In the present study, formation of such small nanoclusters was proposed due to the presence of hyperfine hysteresis structure, while the silanol interaction with MnFe_2_O_4_ tended to decrease the magnetic saturation value [23].

The textural surface properties of spherical silica and MnFe_2_O_4_/silica analyzed using the nitrogen adsorption technique are shown in Figure 1C (a and b), while that of GO and MnFe_2_O_4_/GO are shown in Figure 1D (c and d). silica exhibited a type IV isotherm, where capillary condensation occurred at a high relative pressure of p/p_0_ > 0.8. Loading of manganese spinel ferrite over the silica support significantly reduced the surface area from 170 m^2^/g to 21 m^2^/g. The pore volume was reduced from 0.35 cc/g to 0.1 cc/g, while pore size distribution was reduced from 8.3 to 18.5 nm. The textural surface area reduction of 87%, pore volume of 71% and increase in pore size showed nanodistribution of ferrite nanoparticles filling into the spherical pores of silica and aggregation at the external pore surface. In case of GO, the surface area of the pure GO of 450 m^2^/g was reduced to 186 m^2^/g after spinel impregnation, which exhibited a type IV isotherm curve. Furthermore, the pore volume was reduced from 0.45 cm^3^/g to 0.31 cm^3^/g, and the pore size distribution was reduced from 18.5 nm to 6.6 nm. Our results confirm that our silica and GO nanocomposites were loaded with MnFe_2_O_4_.

**Figure 2:**
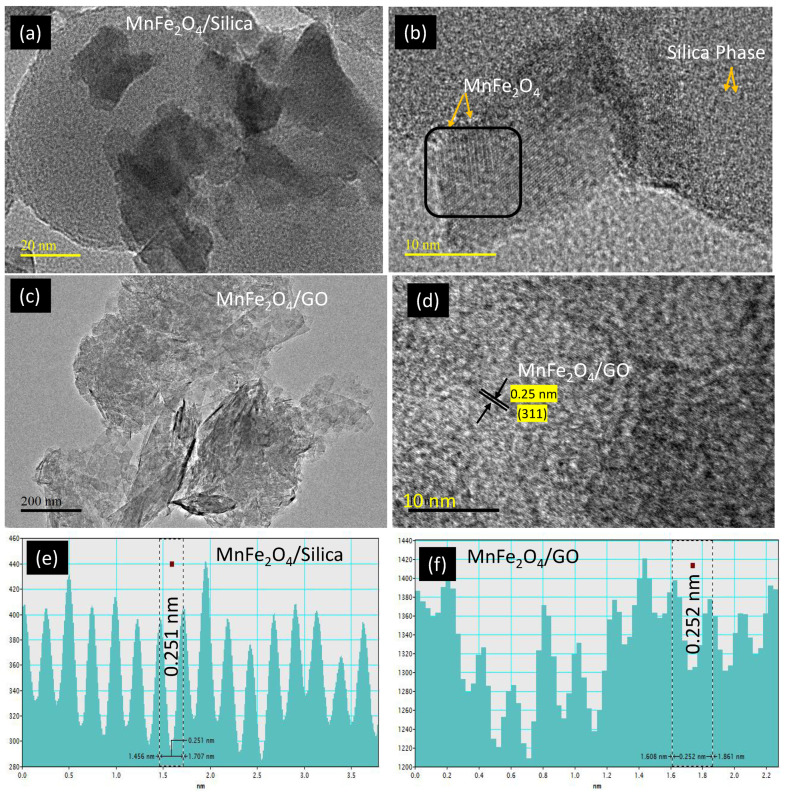
TEM analysis of (**a**,**b**) MnFe_2_O_4_/silica; and (**c**,**d**) MnFe_2_O_4_/GO at different scale magnifications with corresponding lattice fridges (**e**,**f**).

The morphological difference of MnFe_2_O_4_ loading over silica and graphene was analyzed using high resolution transmission electron microscope (HRTEM) (Figure 2a–d). The spherical silica was uniformly dispersed with a diameter of 80 nm, while spinel ferrite nanoparticles (10–20 nm) dispersed across thessilica (Figure 2a,b). The graphene oxide is presented with a thick sheet texture in its un-agglomerated form [24] (Figure 2c,d). The lattice fringes with interplanar distance of 0.25 nm corresponding to (311) plane measured over nanoparticle confirmed the presence of cubic MnFe_2_O_4_ over the two different supports.

The optical response of MnFe_2_O_4_/silica and MnFe_2_O_4_/GO nanocomposites were characterized by diffuse reflectance spectroscopy (Figure S1a,b). The spectra showed a weak absorption below 230 nm and a strong and broad absorption between 240–800 nm. The peaks of MnFe_2_O_4_/silica and MnFe_2_O_4_/GO correlate with cubic spinel exhibits tetrahedral (215 nm) and octahedral (440–700 nm) crystalline coordination sites [25]. The presence of such peak absorption indicates the dispersion and integrated spinel ferrites over graphene oxide support [26]. In the case of GO support, a peak absorbance between 200–230 nm shows the presence of π-π* and n-π* transition due to carbon–carbon and carbonyl linkage in graphene oxide. In addition, an enhanced intense broad peak shows the presence of a higher crystalline mixed phase of oxides due to octahedral coordinated spinel species compared to the silica support (Figure S1a,b).

### 3.2. Drug Delivery Study

**Figure 3:**
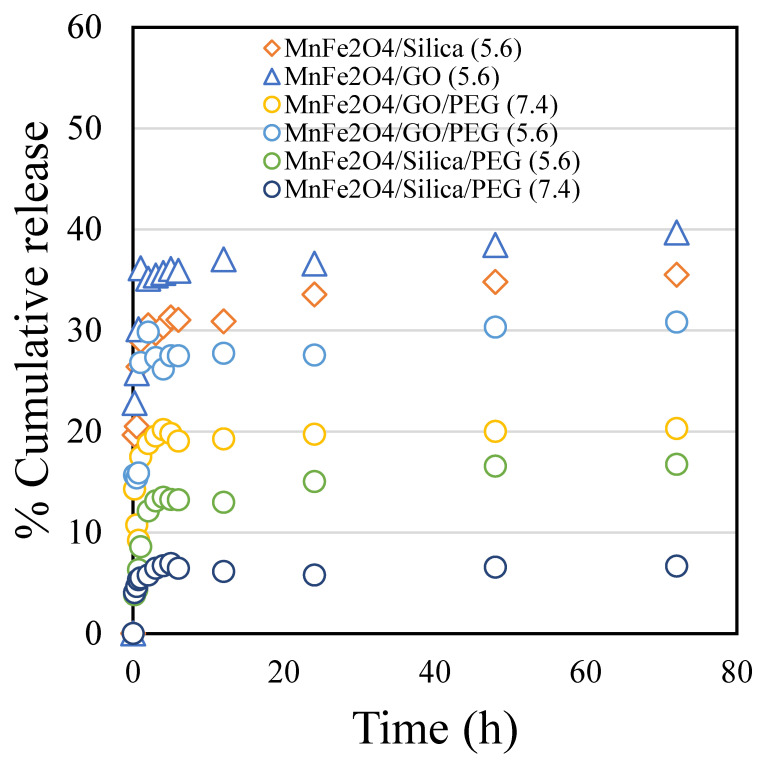
The percentage cumulative cisplatin release in pH 5.6 and 7.4 conditions for 72 h from: MnFe_2_O_4_/silica pH 5.6 (orange diamond); MnFe_2_O_4_/GO pH 5.6 (blue triangle); MnFe_2_O_4_/GO/PEG pH 7.4 (yellow circle); MnFe_2_O_4_/GO/PEG pH 5.6 (blue circle); MnFe_2_O_4_/silica/PEG pH 5.6 (green circle); and MnFe_2_O_4_/silica/PEG pH 7.4 (dark blue circle).

The drug release trend of cisplatin was studied on raw MnFe_2_O_4_/silica, MnFe_2_O_4_/GO and pegylated nanoformulations at pH 5.6 and 7.4 (Figure 3). We found that cisplatin release from both nanocomposites was higher in the acidic environment favoring drug release in the tumor microenvironment. Furthermore, we analyzed the drug release from MnFe_2_O_4_/silica and MnFe_2_O_4_/GO nanocomposites without the PEG coating at pH 5.6 (orange diamond and blue triangle). The cisplatin release was fairly similar with a slight increase in the case of GO. We then tested the drug release from PEG-coated nanocomposites at a different pH. Studying the cisplatin release at pH 7.4 from MnFe_2_O_4_/silica/PEG and MnFe_2_O_4_/GO/PEG nanocomposites (Figure 3, dark blue and yellow circles), we found that the GO nanocomposites resulted in a higher drug release than that seen in silica. In our previous study, we prepared CuFe_2_O_4_/silica, characterized and studied the drug release pattern of cisplatin. The percentage cumulative release of cisplatin from the CuFe_2_O_4_/silica reached to about 80% at 72 h [14]_._ The presence of polyethylene glycol (PEG), a biocompatible hydrophilic polymer, on the surface of GO and silica nanocarriers exhibited a pH sensitive cisplatin release mechanism. At an acidic pH, the polymer layer is reported to undergo hydration and favors high drug/protein release [27]. In our case, the high cisplatin release at pH 5.6 (mimicking the acidic tumor environment) is advantageous over the cisplatin release at pH 7.4, which is the normal physiological pH (i.e., healthy cancer-free cellular environment).

After 72 h, the drug release from the two composites was comparable with 36% release from the silica support and 40% from the GO support. Interestingly, the first two hours showed a slight difference between the two nanocarriers. While the silica nanocarrier showed a steady and controlled drug release, the GO nanocarrier showed a quick release reaching about 36% within 2 h. The loading over manganese spinel ferrite was demonstrated as a reduction in surface area and occupation inside the pores of both supports. However, the slow and steady release in the silica support was better than that in the GO support.

### 3.3. Theoretical Calculation of Cisplatin Interaction with silica and GO

**Figure 4:**
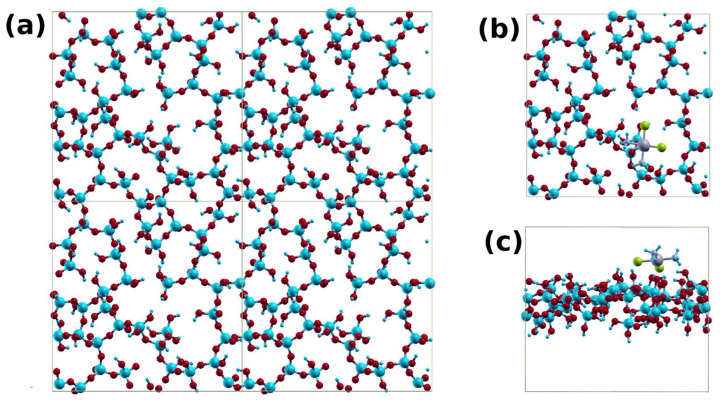
(**a**) A 2 × 2 supercell of the silica system; (**b**) top; and (**c**) side views of the cisplatin–silica structure.

To help interpret our results, we used first-principles calculations to compare cisplatin’s binding on silica and GO. We modeled the case of silica using a slab structure made of 205 atoms in a unit cell of dimensions 22 × 22 × 19 Å (Figure 4a). The thickness of the silica slab was about 7 Å. The slab surface oxygen atoms are properly terminated with H atoms. The cisplatin is initially placed at about 3 Å above the slab surface, and then the system is structurally relaxed (Figure 4b,c). The interaction of the cisplatin with the silica surface is primarily electrostatic. It arises from the H-O and Cl-H interactions between the cisplatin and the silica surface. The shortest H-O and Cl-H distances were 1.78 and 2.14 Å, respectively. The binding energy between cisplatin and silica was found to be 1.5 eV.

**Figure 5:**
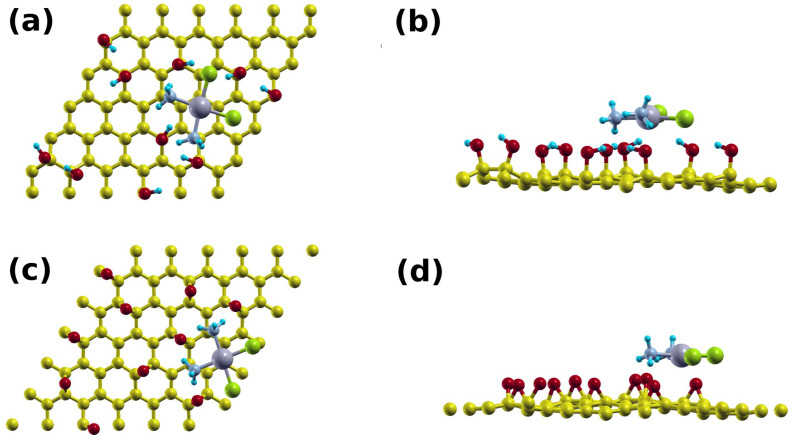
Supercell of the: (**a**) top; and (**b**) side view of the cisplatin-GO1 system, and (**c**) top and (**d**) side views of the cisplatin-GO2 system.

Cisplatin can bind to a single layer or multilayer GO structure. With the latter, cisplatin can further intercalate between the layers, thus creating a *sandwich* structure. We modeled this system using 6 × 6 GO supercell, with two limiting GO models, one with hydroxyl groups (GO1) and the other with epoxy groups (GO2). Structures with cisplatin on single layer GO are shown in Figure 5. We placed the drug about 3 Å above each GO layer. A few electrostatic H bonds were formed between the H atoms of the cisplatin and the hydroxyl groups of GO1 (Figure 5a,b) and the epoxy groups of GO2 (Figure 5c,d), resulting in average bonds lengths of about 1.9 and 2.0 Å, respectively. This resulted in binding energies of 1.47 eV and 1.46 eV, respectively.

**Figure 6:**
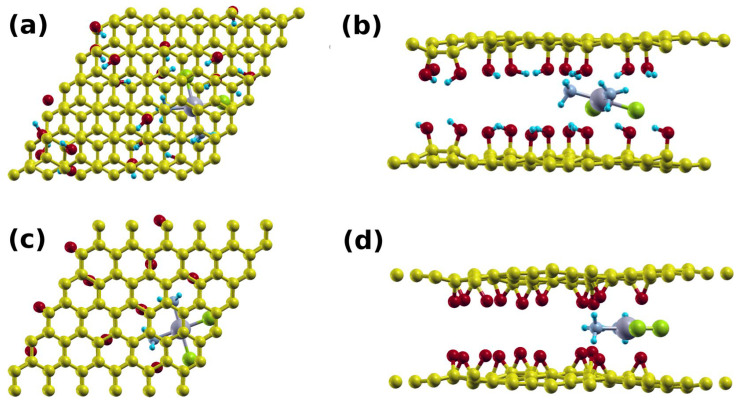
Supercell of the (**a**) top view; and (**b**) side view of the cisplatin double layer GO1 system; and (**c**) top view and (**d**) side view of the cisplatin double layer GO2 system.

The sandwich cisplatin–GO structures are shown in Figure 6 (2GO). The intercalation of the cisplatin established H-bonds with the top and bottom layers, causing the interlayer distances to decrease from 9.7 and 9.5 Å to 8.0 Å and 6.5 Å for GO1 (Figure 6a,b) and GO2 (Figure 6c,d), respectively. We found that the average bond lengths were 1.9 Å and 2.0 Å, and the binding energies were 4.01 eV and 3.02 eV, respectively. The higher binding energies in the sandwich structures are expected as the cisplatin interacts with the top and bottom layers. This leads to higher retention of cisplatin and lower drug release.

### 3.4. In Vitro Cytotoxicity Studies

**Figure 7:**
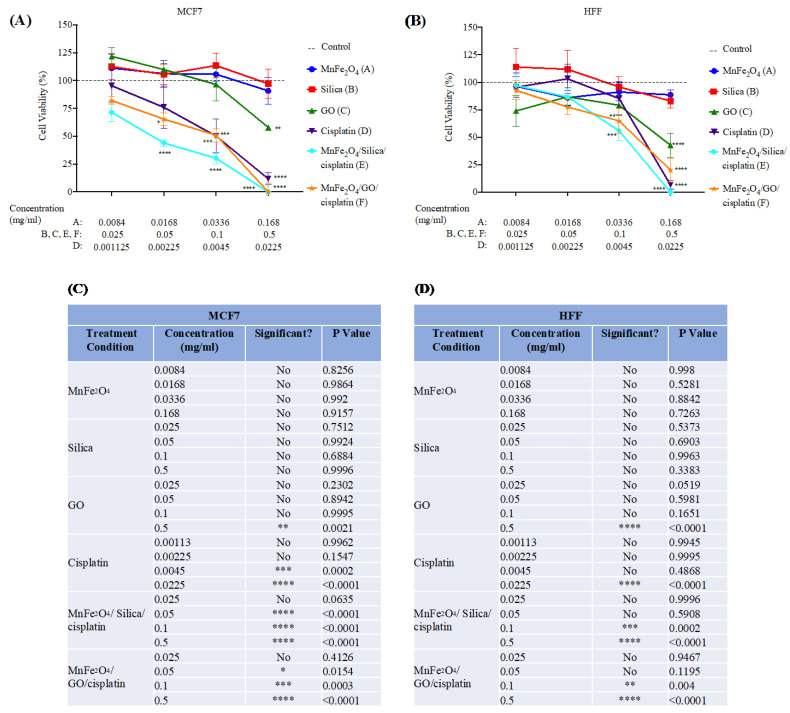
MTT cell viability assay on: (**A**) MCF7; and (**B**) HFF cell lines. Cells were treated with the following conditions for 48 h: MnFe_2_O_4_, silica, GO, Cisplatin, Cisplatin/silica/MnFe_2_O_4_ and Cisplatin/GO/MnFe_2_O_4_; (**C**,**D**) treatment concentrations and statistical analysis for MCF7 (**C**); and HFF (**D**). Different treatment concentrations were used for MnFe_2_O_4_ and cisplatin to reflect the actual concentration adsorbed on the nanocomposite. For details, please see the Materials and Methods section. N = 4 independent experiments. Dashed line represents untreated cells, control. * *p* < 0.05; ** *p* < 0.01; *** *p* < 0.001; **** *p* < 0.0001 versus control using two-way ANOVA with Dunnett’s *post hoc* testing.

To test the cytotoxic effects of our nanocomposites, we used the cell viability assay MTT on MCF7 (human breast cancer cell line), HFF (human foreskin fibroblasts) (Figure 7), HeLa (human cervical adenocarcinoma), HCT116 (human colorectal cancer), and HEK293 (human embryonic kidney) cell lines (Figures S3–S5). Our results showed that the MnFe_2_O_4_ and the silica groups acted as non-cytotoxic agents, the GO only affected cell viability at high concentrations. Interestingly, others have also shown that GO can reduce the cell viability at high concentrations (above 50 µg/mL), but not at lower concentrations [28,29,30]. Moreover, GO was found to result in increased levels of reactive oxygen species (ROS), increased lactate dehydrogenase (LDH), decreased glutathione (GSH), and loss of mitochondrial membrane potential [28,29,30,31,32,33]. These reports suggested that high concentrations of GO can induce mitochondrial damage, oxidative stress, and thus induce apoptosis.

In contrast, treatment with MnFe_2_O_4_/silica/cisplatin, and MnFe_2_O_4_/GO/cisplatin resulted in a significant reduction in cell viability in both cell lines. This reduction was comparable to cells treated with cisplatin (positive control). However, the effect was more pronounced on MCF7 indicated by the half maximal effective concentration (EC50) values, which were lower in MCF7 than in HFF (Figure S2). EC50 for MnFe_2_O_4_/silica/cisplatin, and MnFe_2_O_4_/GO/cisplatin on MCF7 cell line were: 48.43 µg/mL and 85.36 µg/mL, respectively (Figure S2C). EC50 for the same conditions on the HFF cell line were: 102.92 µg/mL and 102.21 µg/mL, respectively (Figure S2D). Our results suggest that the breast cancer cell line, MCF7, is more susceptible to cytotoxic effects by MnFe_2_O_4_/silica/cisplatin and MnFe_2_O_4_/GO/cisplatin. However, the MnFe_2_O_4_/silica/cisplatin nanocomposites seemed to have a more potent toxic effect than the MnFe_2_O_4_/GO/cisplatin nanocomposites. Similar results were observed with other cell lines (Figures S3–S5). HeLa, HCT116, and HEK293 cells were affected by treatment with our nanocomposites in a dose-dependent manner parallel to pure cisplatin. If we analyze dose 2 of treatment with MnFe_2_O_4_/silica/cisplatin, we find that the cell viability was as follows: MCF7 43.98%; HCT116 64.46%; HeLa 56%; HEK293 61.42%; and HFF 86.75%. Moreover, if we analyze the same dose with MnFe_2_O_4_/GO/cisplatin, cell viability was: MCF7 65.37%; HCT116 56.2%; HeLa 55.68%; HEK293 73.24%; and HFF 77.55%. These results show that MCF7 had the lowest cell viability after treatment with a small dose of MnFe_2_O_4_/silica/cisplatin, while HCT116 (cancerous), and HEK293 and HFF (non-cancerous) had the highest cell viability. Furthermore, treatment with a small dose of MnFe_2_O_4_/GO/cisplatin resulted in the lowest viability in HCT116 and HeLa, while HEK293 and HFF had the highest viability, suggesting that there might be a cell-dependent effect.

We previously studied CuFe_2_O_4_/HYPS/cisplatin nanoparticles on MCF7 [14]. The EC50 of CuFe_2_O_4_/HYPS/cisplatin nanoparticles was 180.89 µg/mL, which was 3.7-folds higher than MnFe_2_O_4_/silica/cisplatin (48.43 µg/mL), and two-fold higher than MnFe_2_O_4_/GO/cisplatin (85.36 µg/mL). If we compare the cell viability results at 0.1 mg/mL as an example: cells treated with CuFe_2_O_4_/HYPS/cisplatin had a 48% viability, and MnFe_2_O_4_/GO/cisplatin had a 51% viability. However, MnFe_2_O_4_/silica/cisplatin had a 30% cell viability at the same concentration. These results show that using Pt^4+^/Mn^2+^ as a component in the spinel ferrite nanocomposite on a silica support had a better effect on the cytotoxic capability when compared to Cu^2+^ or Mn^2+^ with GO as a support structure.

**Figure 8:**
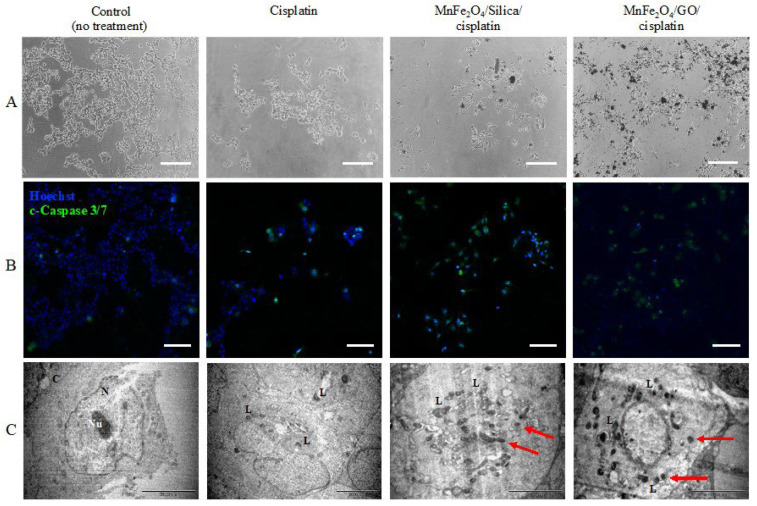
Apoptotic effect of different treatment conditions on MCF7. Cells were treated with cisplatin, MnFe_2_O_4_/silica/cisplatin, and MnFe_2_O_4_/GO/cisplatin for 48 h. The highest dose of each condition was used as follows: cisplatin was 0.0225 mg/mL; MnFe_2_O_4_/silica/cisplatin, and MnFe_2_O_4_/GO/cisplatin was 0.5 mg/m: (**A**) brightfield images (scale bar is 200 µm); (**B**) immunofluorescence images of cells stained with cleaved-Caspase 3/7 (green) and Hoechst (blue) (scale bar is 100 µm); and (**C**) TEM images of treated cells (scale bar is 5 µm). N: nucleus; Nu: nucleolus; Cy: cytoplasm; L: lysosome vacuoles: red arrow: Nanoparticle penetration. Images in A and B are not from the same field of view.

Looking at the brightfield images (Figure 8A), a higher number of cells seem to have been detached from the surface as a result of the treatment with cisplatin, MnFe_2_O_4_/silica/cisplatin, and MnFe_2_O_4_/GO/cisplatin nanocomposites. Apoptotic effects were assessed in the treated cells using the apoptosis hallmarks, c-Caspase 3 and 7 (Figure 8B). To investigate these effects, we used the dual stain c-Caspase 3/7 (green) and the nuclear stain Hoechst (blue). The untreated control cells had a small number of positive c-Caspase 3/7 cells compared to the cells treated with cisplatin (positive control). Similarly, there was a higher number of positive c-Caspase 3/7 cells after treatment with MnFe_2_O_4_/silica/cisplatin, and MnFe_2_O_4_/GO/cisplatin nanocomposites. Despite fluorescence interference of GO [34], the apoptotic effects on cells treated with the MnFe_2_O_4_/GO/cisplatin nanocomposites and MnFe_2_O_4_/silica/cisplatin nanocomposites were clearly seen (red arrow). TEM images were used to investigate the ultrastructural changes in cells treated with our nanocomposites (Figure 8C). The control untreated cells had a regular size nucleus with normal ultrastructure [35]. However, cisplatin-treated cells had an increased number of lysosome vacuoles and a higher number in those treated with MnFe_2_O_4_/silica/cisplatin and MnFe_2_O_4_/GO/cisplatin. Our findings from the immunofluorescent staining and TEM images indicate that cells treated with MnFe_2_O_4_/silica/cisplatin, and MnFe_2_O_4_/GO/cisplatin nanocomposites resulted in apoptosis as the major mechanism of cell death.

Finally, to further investigate whether our NPs resulted in necrosis in addition to apoptosis, we performed an AV/PI analysis on cells treated with MnFe_2_O_4_/silica/cisplatin and MnFe_2_O_4_/GO/cisplatin nanocomposites at a concentration of 0.05 mg/mL (dose 2) for 6 and 24 h. Our results (Figure S6) showed that treatment with these nanocomposites resulted in an increased signal intensity of AV and a modest increase in the PI compared to the no-treatment control. In parallel to the MTT results of HCT116 (Figure S4), dose 2 resulted in a higher cytotoxicity after treatment with MnFe_2_O_4_/GO/cisplatin (56.2%) than with MnFe_2_O_4_/silica/cisplatin (64.46%). These results suggest an early apoptosis (AV+/PI−) at 6 h and the start of a late apoptosis or a secondary necrosis as indicated by AV+ and PI+ at 24 h [20]. However, these results need to be confirmed in future work by investigating protein activation and the signaling pathways involved in the mechanism.

Manganese ferrite nanoparticles were impregnated over silica and GO supports. The nanocomposites were characterized using various physico-chemical techniques. The cisplatin drug release study was performed using a dialysis technique (Figure 1, Figure 2 and Figure 3). As observed in the TEM images (Figure 2a–c), silica nanoparticles possessed a monodispersed configuration. With that assumption, the calculations revealed that the interaction between cisplatin and silica resulted in a binding energy equal to 1.5 eV. On the other hand, to accurately calculate the binding energy of GO that presented with a sheet-like structure, we assumed three scenarios: (1) a single layer with cisplatin binding on top and bottom; (2) agglomerated multiple layers that will bind with cisplatin as a single layer; and (3) multiple layers with cisplatin interspersed within the layers. The first case (single layer) resulted in a binding energy of 1.5 eV, which was equal to the energy required to bind cisplatin to silica. The second and third scenarios will result in a binding energy equal to 3–4 eV. The third case (cisplatin interspersed between multiple layers) will result in lower release of cisplatin. In an acidic environment, the H^+^ ions will diffuse between the layers and bind with the OH- group in GO resulting in the formation of H_2_O, thus uncoupling of the GO layers, releasing the cisplatin. However, our drug release studies (Figure 3, orange diamond and blue triangle) suggest that it is more likely that GO behaves as a single layer, where cisplatin binds to the top and bottom of each layer with a binding energy of 1.5 eV.

We found that the drug release from both pegylated nanocomposites (MnFe_2_O_4_/silica/PEG and MnFe_2_O_4_/GO/PEG) was higher in the acidic environment favoring drug release in the tumor microenvironment. Furthermore, the drug release from raw nanoformulations (MnFe_2_O_4_/silica and MnFe_2_O_4_/GO) without the PEG coating at pH 5.6 (orange diamond and blue triangle) shows a fairly similar trend with a slight increase in cisplatin release than GO. In the case of PEG-coated nanocomposites at different pH, (Figure 3, dark blue and yellow circles), MnFe_2_O_4_/GO/PEG nanocomposite exhibited a higher drug release than that seen in MnFe_2_O_4_/silica/PEG. These results support the assumption that cisplatin intercalates between the GO sheets. Moreover, drug release at pH 5.6 (Figure 3, green and light blue circles), showed that GO nanocomposites resulted in a higher release than silica. It might be argued that the increased drug release from GO nanocomposites in both pH values was probably due to an inefficient PEG wrapping with limited functional moieties of GO at the edges (5%), but also the hydrogen bonds between silica and PEG break in acidic conditions leading to enhanced drug release. However, even in the absence of PEG wrapping (Figure 3, blue triangle, and orange diamond), the GO nanocomposites had a slightly higher cisplatin release than silica. Taken together, our cisplatin release studies suggest that while our nanocomposites had a similar release without the PEG coating, MnFe_2_O_4_/GO/PEG nanocomposites had a higher release than MnFe_2_O_4_/silica/PEG in both neutral and acidic environment. We calculated the binding energy between cisplatin–silica and cisplatin–GO using the DFT calculations (Figure 4, Figure 5 and Figure 6). However, our cytotoxicity analysis revealed that our nanocomposites had similar (Figure 7—HFF) if not higher cytotoxicity with MnFe_2_O_4_/silica/cisplatin (Figure 7—MCF7). This might be explained by the size and shape of these nanocomposites. While MnFe_2_O_4_/silica presented as uniform spheres with a surface area of 21 m^2^/g, MnFe_2_O_4_/GO nanocomposites appeared as nanowire sheets with a surface area of 186 m^2^/g (Figure 1). The smaller and more uniform shape of the MnFe_2_O_4_/silica nanocomposites may have enhanced their cellular internalization and cisplatin delivery. Thus, the proposed mechanism of delivery in MnFe_2_O_4_/silica is either through: 1) the release of cisplatin, which is then taken up by cells, or 2) cellular internalization of the MnFe_2_O_4_/silica nanocomposite. On the other hand, due to the irregular shape and size of the MnFe_2_O_4_/GO nanocomposites, the most likely mechanism is through the release of cisplatin that is bound to the top and bottom of the GO sheets, thus reducing the efficiency of cisplatin delivery.

In line with our results, researchers have reported that GO can cause a reduction in viability in a dose-dependent manner [36,37]_._ It has been shown that GO was more cytotoxic than its non-oxidize graphene form and toxicity increases with oxidation [37]. Cytotoxicity was also affected by the particle size, with the smaller-sized NPs being more potent. It was reported that the reduced cell viability was most likely attributed to the oxidative stress and apoptosis [29]. In addition, doxorubicin-loaded graphene oxide MnFe_2_O_4_ nanoparticles had an increased release of doxorubicin and a decreased cell viability when subjected to laser irradiation [38].

Although our results show that the cytotoxicity of silica was higher than that of GO, we believe that GO is a better drug carrier. Our assumption is based on the multi-layer structure of GO allowing for the intercalation of cisplatin resulting in a higher loading capacity. This can be best utilized if the GO particles are made small enough that they can easily penetrate the cancer cells. As demonstrated by other groups [39], stacked graphite nanofibers that were incorporated with cisplatin had a cytotoxicity higher than the free drug. In addition, as we observed in our results (Figure 3 and Figure 6), the strong binding energy between cisplatin and GO might allow for a slow and sustained release over a longer duration [40]. Moreover, GO NPs are capable of assuming a 3D spherical structure (90–120 nm in diameter) when loaded with cisplatin [41]. After cellular internalization and drug release, they can revert to their 2D structure. Furthermore, it is feasible to design multi-drug loaded PEGylated GO [8]. These nanocomposites showed minimal systemic toxicity to other organs (such as the heart and kidneys) when compared to treatment with the free drugs. These findings provide a hint of the versatility and multiple applications of GO making it a strong candidate for future research. Taken together, our results suggest that while MnFe_2_O_4_/silica/cisplatin nanocomposites might seem like a better chemotherapeutic option than MnFe_2_O_4_/GO/cisplatin nanocomposites. A decrease in the size of the GO particles may lead to a superior performance.

## 4. Conclusions

Here, we designed and compared the efficiency of MnFe_2_O_4_/silica/cisplatin and MnFe_2_O_4_/GO/cisplatin nanoformulations as a novel chemotherapeutic option. Characterization studies confirmed the cubic spinel particle dispersion of about 10–20 nm in tetrahedral and octahedral crystalline coordination sites. The drug release study demonstrated a slower and steadier release of cisplatin from the silica support than the GO support. We used density functional theory to calculate the binding energies between cisplatin and both silica and graphene oxide. The cisplatin binding energy to silica was 1.5 eV. In the case of GO, we assumed a single layer or multilayer (sandwich) GO structure with two limiting groups: hydroxyl groups (GO1) and epoxy groups (GO2). For the single layer GO structure, the binding energies with cisplatin were 1.47 eV (GO1) and 1.46 eV (GO2). For the sandwich GO structure, the binding energies were 4.01 eV (GO1) and 3.02 eV (GO2). MnFe_2_O_4_/silica/cisplatin and MnFe_2_O_4_/GO/cisplatin treated MCF7 (breast cancer cells) and HFF (human foreskin fibroblast) with these nanocomposites resulted in a significant reduction in cell viability. The EC50 for MnFe_2_O_4_/silica/cisplatin and MnFe_2_O_4_/GO/cisplatin on MCF7 was: 48.43 µg/mL and 85.36 µg/mL, respectively. EC50 for the same conditions on HFF was: 102.92 µg/mL and 102.21 µg/mL, respectively. Our results suggest that the breast cancer cell line, MCF7, is more susceptible to cytotoxic effects by MnFe_2_O_4_/silica/cisplatin and MnFe_2_O_4_/GO/cisplatin. Immunofluorescence images using c-caspase 3/7 and TEM analysis indicated that these nanocomposites resulted in apoptosis as the major mechanism of cell death. Thus, the results show that MnFe_2_O_4_/silica/cisplatin and MnFe_2_O_4_/GO/cisplatin nanocomposites are interesting novel chemotherapeutic options for breast cancer with the added advantage of the possibility of magnetically guided delivery, controlled release (by laser irradiation), and imaging.

## Data Availability

The obtained data in this study is provided in this manuscript and in supplementary file. Further additional data can be provided from corresponding authors upon request.

## Supplementary Materials

The following supporting information can be downloaded at: https://www.mdpi.com/article/10.3390/cancers15030695/s1, **Figure S1**. Diffuse reflectance UV-Visible spectra of (a) MnFe_2_O_4_/silica and (b) MnFe_2_O_4_/GO; **Figure S2**: EC50 analysis. (A,B) Data from Figure 8 were used to extrapolate the line equation of: MnFe_2_O_4_, silica, GO, cisplatin, MnFe_2_O_4_/silica/cisplatin, and MnFe_2_O_4_/GO/cisplatin. (A) represents data from MCF7, and (B) HFF cell lines. Line equations were used to calculate the EC50 for each nanocomposite tested on MCF7 (C) and HFF (D) cell lines; **Figure S3**: (A) MTT cell viability assay on HeLa cell line. Cells were treated with the following conditions for 48 h: MnFe_2_O_4_, silica, GO, cisplatin, MnFe_2_O_4_/silica/cisplatin and MnFe_2_O_4_/GO/cisplatin. (B) Treatment concentrations and statistical analysis. Different concentrations were used for MnFe_2_O_4_ and cisplatin to reflect the actual concentration adsorbed on the nanocomposite. For details, please see the Materials and Methods section. n= 3 independent experiments. Dashed line represents untreated cells, control. * *p* < 0.05; ** *p* < 0.01; *** *p* < 0.001; **** *p* < 0.0001 versus control using two-way ANOVA with Dunnett’s post hoc testing; **Figure S4**: (A) MTT cell viability assay on HCT116 cell line. Cells were treated with the following conditions for 48h: MnFe_2_O_4_, silica, GO, Cisplatin, MnFe_2_O_4_/silica/cisplatin and MnFe_2_O_4_/GO/cisplatin. (B) Treatment concentrations and statistical analysis. Different concentrations were used for MnFe_2_O_4_ and cisplatin to reflect the actual concentration adsorbed on the nanocomposite. For details, please see the Materials and Methods section. n= 3 independent experiments. Dashed line represents untreated cells, control. * *p* < 0.05; ** *p* < 0.01; *** *p* < 0.001; **** *p* < 0.0001 versus control using two-way ANOVA with Dunnett’s post hoc testing; **Figure S5**: (A) MTT cell viability assay on HEK293 cell line. Cells were treated with the following conditions for 48 h: MnFe_2_O_4_, silica, GO, Cisplatin, MnFe_2_O_4_/silica/cisplatin and MnFe_2_O_4_/GO/cisplatin. (B) Treatment concentrations and statistical analysis. Different concentrations were used for MnFe_2_O_4_ and cisplatin to reflect the actual concentration adsorbed on the nanocomposite. For details, please see the Materials and Methods section. n = 3 independent experiments. Dashed line represents untreated cells, control. * *p* < 0.05; ** *p* < 0.01; *** *p* < 0.001; **** *p* < 0.0001 versus control using two-way ANOVA with Dunnett’s post hoc testing; **Figure S6**: HCT116 cells were treated with 0.05 mg/mL of MnFe_2_O_4_/silica/cisplatin and MnFe_2_O_4_/GO/cisplatin and its equivalent concentration of cisplatin (0.00225 mg/mL for details, please check the Materials and Methods section). Cells were treated for 6 h (A), and 24 h (B) and then stained with Annexin V (AV), Propidium Iodide (PI), and Hoechst. n = 5 independent experiments.
