# Supplementary material for: Insights of Platinum Drug Interaction with Spinel Magnetic Nanocomposites for Targeted Anti-Cancer Effect
Source: Cancers (Basel). 2023 Jan 23;15(3):695. doi: 10.3390/cancers15030695 (PMC9913461; doi:10.3390/cancers15030695)
Supplement: Supplementary file 1 [file cancers-15-00695-s001.zip › cancers-2015204-supplementary.pdf]

Supplementary File

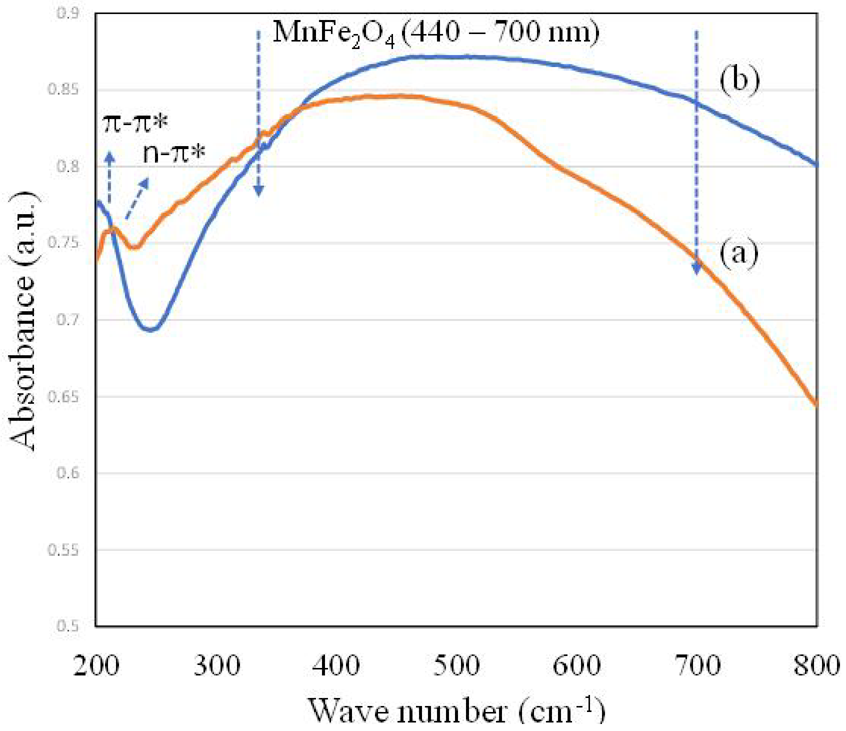

Figure S1. Diffuse reflectance UV-Visible spectra of (a) $MnFe_2O_4$/Silica and (b) $MnFe_2O_4$/GO.

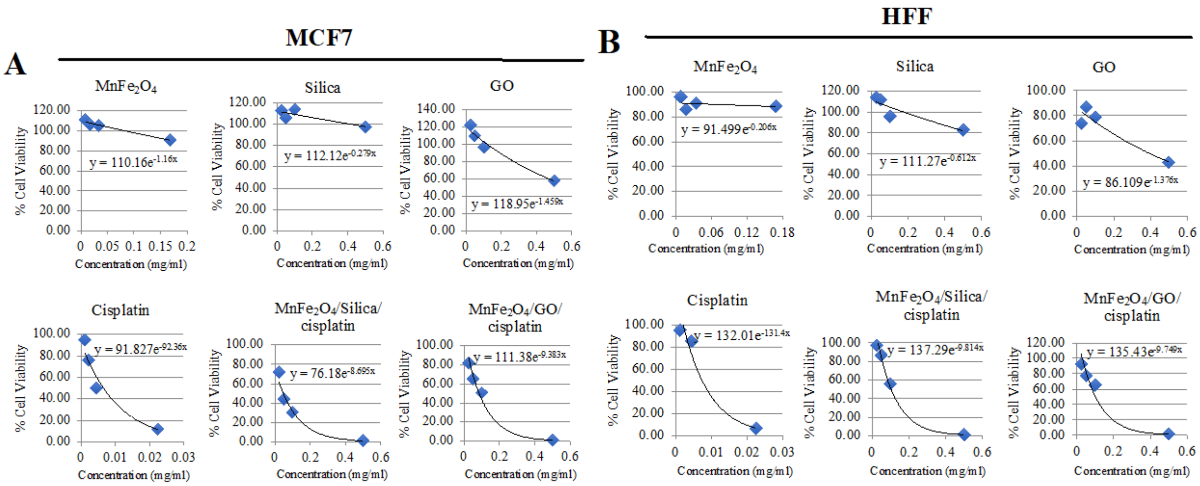

C

Drug Group	Equation	EC50 value (mg/ml)
$MnFe_2O_4$	$y = 110.16e^{-1.16x}$	0.68096
Silica	$y = 112.12e^{-0.279x}$	2.89443
GO	$y = 118.95e^{-1.459x}$	0.59402
Cisplatin	$y = 91.827e^{-92.36x}$	0.00658
$MnFe_2O_4$/Silica/cisplatin	$y = 76.18e^{-8.695x}$	0.04843
$MnFe_2O_4$/GO/cisplatin	$y = 111.38e^{-9.383x}$	0.08536

D

Drug Group	Equation	EC50 value (mg/ml)
$MnFe_2O_4$	$y = 91.499e^{-0.206x}$	2.93352
Silica	$y = 111.27e^{-0.612x}$	1.30709
GO	$y = 86.109e^{-1.376x}$	0.39505
Cisplatin	$y = 132.01e^{-131.4x}$	0.00739
$MnFe_2O_4$/Silica/cisplatin	$y = 137.29e^{-9.814x}$	0.10292
$MnFe_2O_4$/GO/cisplatin	$y = 135.43e^{-9.749x}$	0.10221

Figure S2: EC50 analysis. (**A**, **B**) Data from Fig. 8 were used to extrapolate the line equation of: $MnFe_2O_4$, silica, GO, cisplatin, $MnFe_2O_4$/silica/cisplatin, and $MnFe_2O_4$/GO/cisplatin. (**A**) represents data from MCF7, and (**B**) HFF cell lines. Line equations were used to calculate the EC50 for each nanocomposite tested on MCF7 (**C**) and HFF (**D**) cell lines.

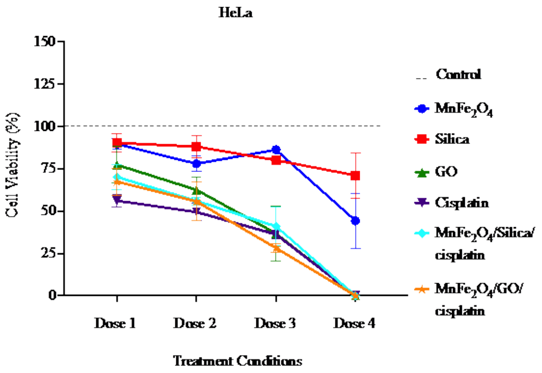

HeLa				
Treatment Condition	Concentration (mg/ml)	Significant ?	P Value	EC50 (mg/ml)
$MnFe_2O_4$	0.0084	No	0.753	0.1485
	0.0168	No	0.101	
	0.0336	No	0.495	
	0.168	****	<0.0001	
Silica	0.025	No	0.799	1.0658
	0.05	No	0.638	
	0.1	No	0.161	
	0.5	*	0.017	
GO	0.025	No	0.088	0.1276
	0.05	**	0.001	
	0.1	****	<0.0001	
	0.5	****	<0.0001	
Cisplatin	0.00113	***	0.0001	0.0016
	0.00225	****	<0.0001	
	0.0045	****	<0.0001	
	0.0225	****	<0.0001	
$MnFe_2O_4$/ Silica/ cisplatin	0.025	*	0.013	0.1062
	0.05	***	0.0001	
	0.1	****	<0.0001	
	0.5	****	<0.0001	
$MnFe_2O_4$/ GO/cisplatin	0.025	**	0.006	0.0692
	0.05	***	0.0001	
	0.1	****	<0.0001	
	0.5	****	<0.0001	

Figure S3: (**A**) MTT cell viability assay on HeLa cell line. Cells were treated with the following conditions for 48h: $MnFe_2O_4$, silica, GO, cisplatin, $MnFe_2O_4$/silica/cisplatin and $MnFe_2O_4$/GO/cisplatin. (**B**) Treatment concentrations and statistical analysis. Different concentrations were used for $MnFe_2O_4$ and cisplatin to reflect the actual concentration adsorbed on the nanocomposite. For details, please see the Materials and Methods section. n= 3 independent experiments. Dashed line represents untreated cells, control. * $p<0.05$; ** $p<0.01$; *** $p<0.001$; **** $p<0.0001$ versus control using two-way ANOVA with Dunnett's *post hoc* testing.

(A)

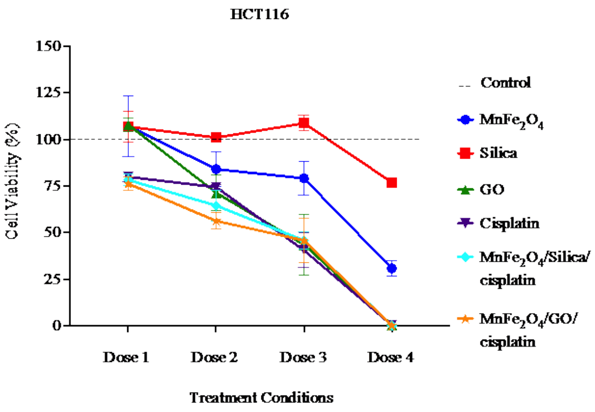

(B)

HCT116				
**Treatment Condition**	**Concentration (mg/ml)**	**Significant ?**	**P Value**	**EC50 (mg/ml)**
$MnFe_2O_4$	0.0084	No	0.932	0.1181
	0.0168	No	0.336	
	0.0336	No	0.124	
	0.168	****	<0.0001	
Silica	0.025	No	0.946	0.9440
	0.05	No	1	
	0.1	No	0.855	
	0.5	No	0.076	
GO	0.025	No	0.902	0.2009
	0.05	*	0.017	
	0.1	****	<0.0001	
	0.5	****	<0.0001	
Cisplatin	0.00113	No	0.148	0.0072
	0.00225	*	0.039	
	0.0045	****	<0.0001	
	0.0225	****	<0.0001	
$MnFe_2O_4$/ Silica/ cisplatin	0.025	No	0.101	0.1492
	0.05	**	0.002	
	0.1	****	<0.0001	
	0.5	****	<0.0001	
$MnFe_2O_4$/ GO/cisplatin	0.025	No	0.062	0.1295
	0.05	***	0.0001	
	0.1	****	<0.0001	
	0.5	****	<0.0001	

Figure S4: (**A**) MTT cell viability assay on HCT116 cell line. Cells were treated with the following conditions for 48h: MnFe2O4, silica, GO, Cisplatin, MnFe2O4/silica/cisplatin and MnFe2O4/GO/cisplatin. (**B**) Treatment concentrations and statistical analysis. Different concentrations were used for MnFe2O4 and cisplatin to reflect the actual concentration adsorbed on the nanocomposite. For details, please see the Materials and Methods section. n= 3 independent experiments. Dashed line represents untreated cells, control. * p<0.05; ** p<0.01; *** p<0.001; **** p<0.0001 versus control using two-way ANOVA with Dunnett's post hoc testing.

(A)

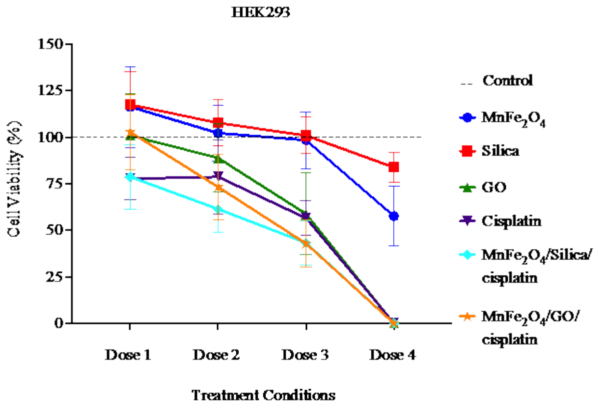

(B)

HEK293				
**Treatment Condition**	**Concentration (mg/ml)**	**Significant ?**	**P Value**	**EC50 (mg/ml)**
$MnFe_2O_4$	0.0084	No	0.512	0.1889
	0.0168	No	0.9997	
	0.0336	No	0.9998	
	0.168	**	0.0025	
Silica	0.025	No	0.4426	1.0553
	0.05	No	0.9607	
	0.1	No	0.9999	
	0.5	No	0.5217	
GO	0.025	No	0.9999	0.2311
	0.05	No	0.8276	
	0.1	**	0.0035	
	0.5	****	<0.0001	
Cisplatin	0.00113	No	0.2156	0.0085
	0.00225	No	0.2532	
	0.0045	**	0.0019	
	0.0225	****	<0.0001	
$MnFe_2O_4$/ Silica/ cisplatin	0.025	No	0.2524	0.1398
	0.05	**	0.0068	
	0.1	****	<0.0001	
	0.5	****	<0.0001	
$MnFe_2O_4$/ GO/cisplatin	0.025	No	0.9996	0.1954
	0.05	No	0.0966	
	0.1	****	<0.0001	
	0.5	****	<0.0001	

Figure S5: (**A**) MTT cell viability assay on HEK293 cell line. Cells were treated with the following conditions for 48h: MnFe2O4, silica, GO, Cisplatin, MnFe2O4/silica/cisplatin and MnFe2O4/GO/cisplatin. (**B**) Treatment concentrations and statistical analysis. Different concentrations were used for MnFe2O4 and cisplatin to reflect the actual concentration adsorbed on the nanocomposite. For details, please see the Materials and Methods section. n= 3 independent experiments. Dashed line represents untreated cells, control. * $p<0.05$; ** $p<0.01$; *** $p<0.001$; **** $p<0.0001$ versus control using two-way ANOVA with Dunnett's post hoc testing.

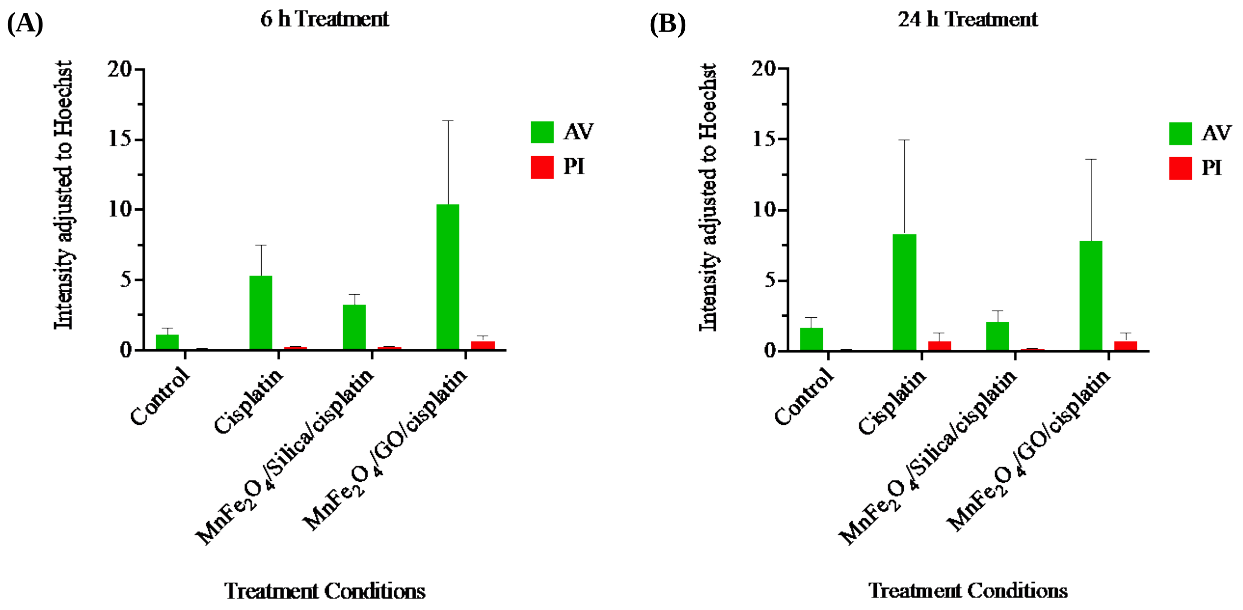

Figure S6: HCT116 cells were treated with 0.05 mg/ml of $MnFe_2O_4$/silica/cisplatin and $MnFe_2O_4$/GO/cisplatin and its equivalent concentration of cisplatin (0.00225 mg/ml for details, please check the Materials and Methods section). Cells were treated for 6 h (**A**), and 24 h (**B**) and then stained with Annexin V (AV), Propidium Iodide (PI), and Hoechst. n= 5 independent experiments.
